# The differential susceptibilities of MCF-7 and MDA-MB-231 cells to the cytotoxic effects of curcumin are associated with the PI3K/Akt-SKP2-Cip/Kips pathway

**DOI:** 10.1186/s12935-014-0126-4

**Published:** 2014-11-30

**Authors:** Tao Jia, Li Zhang, Yale Duan, Min Zhang, Gang Wang, Jun Zhang, Zheng Zhao

**Affiliations:** Key Laboratory of Brain Functional Genomics, Ministry of Education Shanghai Key Laboratory of Brain Functional Genomics, East China Normal University, School of Life Sciences, Shanghai, 200062 China; Department of Clinical Laboratory, Shanghai Public Health Clinical Center Affiliated to Fudan University, Shanghai, 201508 China; Current address: INSERM U823, Grenoble, F-38042 France

**Keywords:** Curcumin sensitivity, Breast cancer, PI3K, SKP2, FOXO, Cip/Kips

## Abstract

**Background:**

The mechanism underlying the differential cytotoxicity of curcumin in various cancer types, however, remains largely unclear. The aims of this study is to examine the concentration- and time-related effects of curcumin on two different breast cancer cells, MCF-7 and MDA-MB-231, and investigated the functional changes induced by curcumin treatment, as well as their relationship to the PI3K/Akt-SKP2-Cip/Kips pathway.

**Methods:**

First, WST-1 and clonogenic assay were performed to determine the cytotoxicity of curcumin in MCF-7 and MDA-MB-231 cells. Then, the expression of CDK interacting protein/Kinase inhibitory protein (Cip/Kips) members (p27, p21 and p57) and S-phase kinase-associated protein-2 (SKP2) was investigated by QRT PCR and Western Blotting. Curcumin’s effect on PI3K (phosphatidylinositol 3-kinase) /Akt and its substrates Foxo1 and Foxo3a were then studied by Western Blotting. Small interfering RNAs (siRNAs) targeting SKP2 was used to explore the relationship between SKP2 and Cip/Kips members. Finally, WST-1 assay was tested to explore the concomitant treatment with curcumin and the inhibition of PKB or SKP2 signaling on curcumin sensitivity in MCF-7 and MDA-MB-231 cells.

**Results:**

We demonstrated MCF-7 and MDA-MB-231 cells exhibited differential responses to curcumin by WST-1 and clonogenic assay (MDA-MB-231 cells was sensitive, and MCF-7 cells was resistant), which were found to be related to the differential curcumin-mediated regulation of SKP2-Cip/Kips (p21 and p27 but not p57) signaling. The differential cellular responses were further linked to the converse effects of curcumin on PI3K/Akt and its substrates Foxo1 and Foxo3a. Importantly, PI3K inhibitor wortmannin could counteract both curcumin-induced phosphorylation of Akt and up-regulation of SKP2 in MCF-7 cells. Subsequent WST-1 assay demonstrated concomitant treatment with curcumin and wortmannin or SKP2 siRNA not only further augmented curcumin sensitivity in MDA-MB-231 cells but also overcame curcumin resistance in MCF-7 cells.

**Conclusions:**

Our study established PI3K/Akt-SKP2-Cip/Kips signaling pathway is involved in the mechanism of action of curcumin and revealed that the discrepant modulation of this pathway by curcumin is responsible for the differential susceptibilities of these two cell types to curcumin.

## Background

Breast cancer remains the most common type of cancer in Asia. Despite displaying similar clinic pathological features, however, patients typically respond differently to therapies and display significantly different outcomes, indicating the substantial diversity of the various cancer subtypes. Indeed, genomic, epigenetic, transcriptional, and proteomic analyses have revealed that breast cancer subtypes may differ in pathway activity, progression, and response to therapy [1]. Thus, a better understanding of the molecular mechanisms that impact patient response and resistance to candidate therapeutics may be helpful in predicting clinical response and developing more effective treatments for different subtypes of breast cancer.

Curcumin is a popular phytochemical that is used as a dietary additive. In Asia, dietary curcumin intake is very high; adults consume up to 200 mg, or 7-8 μmol/kg of body weight, per day [2]. Curcumin has repeatedly been reported to be efficient and safe for the prevention and treatment of a wide spectrum of pathological conditions, including cancer [3,4]. Mounting evidence suggests that the anti-tumor activity of curcumin may be attributed to its ability to induce apoptosis and arrest cell growth via pleiotropic mechanisms [3,5,6]. However, recent studies have also challenged the practice of dietary curcumin supplementation in cancer patients undergoing chemotherapy, including breast cancer patients [7,8], suggesting that it is necessary to first establish the benefit-risk profile of curcumin [9]. Moreover, clinical trial data has indicated that patients respond differently to curcumin, which may be due to the differential molecular signaling that underlies distinct subtypes of cancer.

Considerable evidence exists demonstrating the roles of cell-cycle mediators in determining a cell’s fate toward proliferation, arrest, differentiation, quiescence, or apoptosis. The roles of members of the Cip/Kips family of cyclin-dependent kinase inhibitors (CDKi), such as p21 CIP1/WAF1/CDKN1A, p27 KIP2/CDKN1B, and p57 KIP2/CDKN1C, in negatively regulating the cell cycle and in genomic stability, apoptosis, senescence and DNA repair have been well characterized [10,11]. Their functional changes may be associated with the differential susceptibilities of different cancer subtypes to anticancer drugs. However, the roles of members of the Cip/Kips family (p27, p21, and p57) in mediating cellular responses to curcumin in different subtypes of breast cancer cells remains widely unknown.

SKP2 is the specific substrate-recognition subunit of the SKP1 Cullin-F-box protein (SCF) type ubiquitin ligase complex. SKP2 has emerged as an important player in cell fate decisions by mediating the degradation of specific substrates, including members of the Cip/Kips family (p27, p57, and p21), p130, and c-Myc [12,13]. SKP2 is considered to have strong independent prognostic potential and be a useful target for the treatment of breast cancer [14,15]. SKP2 alterations have also been found to be key mediators that are involved in cancer growth and drug resistance [16-18]. Although the mechanisms that regulate SKP2 protein levels and how the protein differentially modulates the susceptibilities of different breast cancer cell types to chemotherapeutic drugs, such as curcumin, are not clearly understood, the PI3K/Akt survival pathway has been stated to be closely associated with the functions of SKP2. Lin et al. and Gao et al. reported that Akt-mediated phosphorylation of SKP2 at Ser-72 impaired its degradation and re-localized it to the cytosol, contributing to its oncogenic functions [19,20]. In contrast, Boutonnet et al. and Bashir et al. reported that phosphorylation of SKP2 on Ser-72 did not control SKP2 binding to SKP1 and CUL1 and had no influence on SCFSKP2 ubiquitin ligase activity and its subcellular localization [21,22]. Despite these conflicting reports, the results of these studies implied that activation of the PI3K/Akt pathway plays a positive role in stabilizing SKP2 and thereby promoting its oncogenic activities in regulating cell cycle progression, senescence and metastasis. Interestingly, the results of a recent study revealed that the SKP2 SCF complex was a critical E3 ligase for ErbB-receptor-mediated Akt ubiquitination, activation and membrane recruitment, and that SKP2 deficiency inhibited Akt activation and Glut1 expression, leading to repression of breast cancer development [23]. These findings imply that a positive feedback loop composed of SKP2 and Akt promotes cancer cell progression through the restriction point. Furthermore, inhibition of the PI3K/Akt pathway by LY294002 was reported to result in p27 accumulation, which was associated with a decrease in SKP2 levels in MCF-7 cells [24]. However, whether curcumin regulates SKP2 expression via this pathway in breast cancer remains unknown.

The present study aimed to seek the signaling pathway responsible for the differential susceptibilities of two different breast cancer cell lines, MCF-7 and MDA-MB-231, to the cytotoxic effects of curcumin. The concentration- and time-related roles of curcumin in modulating the distinct cellular responses of Cip/Kips proteins (p21, p27 and p57) and their upstream effectors, SKP2 and PI3K/Akt, were systematically examined. The interactions between these signaling molecules were further investigated in an effort to explain why MDA-MB-231 cells were sensitive, whereas MCF-7 cells were resistant, to curcumin treatment. The factors, such as SKP2 and Akt, that governed sensitivity to curcumin, especially in the curcumin-resistant MCF-7 cells, were subsequently evaluated. The results of the present study thus expanded our knowledge of the PI3K/Akt-SKP2-Cip/Kips pathway and highlighted the critical implication of this pathway in overcoming the drug (curcumin) resistance that is commonly observed in different subtypes of breast cancer.

## Results

### The cytotoxicity of curcumin differs in MCF-7 and MDA-MB-231 cells

**Figure 1:**
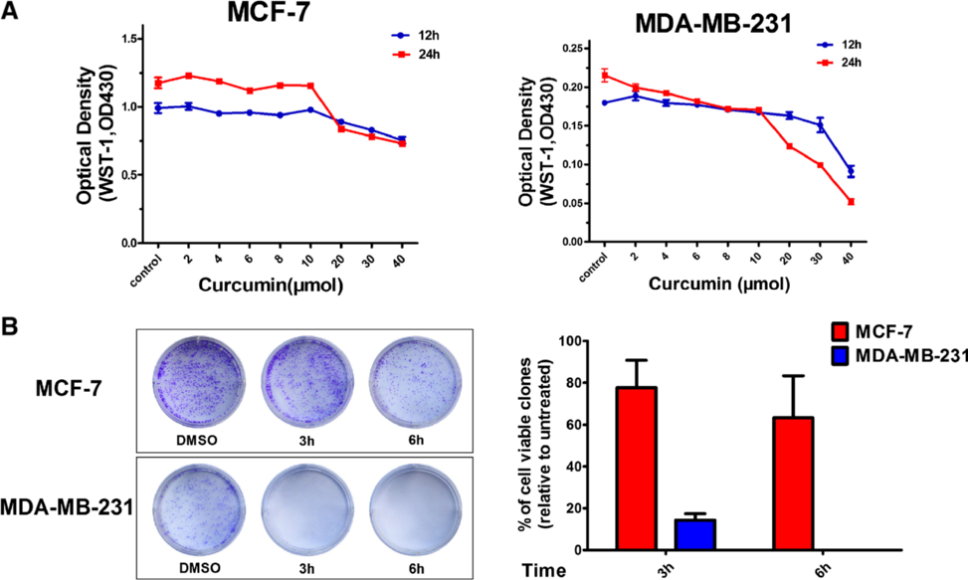
**Curcumin’s responsiveness of MCF-7 and MDA-MB-231 cells. (A)** WST-1 assay of MCF-7 and MDA-MB-231 cells after curcumin treatment. The indicated breast cancer cells were treated with indicated concentrations of curcumin for 12 or 24 h as described in the text. Data represents the fluorescent changes (n = 3). The experiments were performed three times. **(B)** Clonogenic analysis of MCF-7 and MDA-MB-231 cells after curcumin treatment. The indicated breast cancer cells were treated with 40 μM curcumin for 3 or 6 h, trypsinized, and plated in duplicates at low density (2,000 per well in 6 well plate). After 7–12 days, formed colonies were stained with crystal violet. Clones in a given area were counted for each condition. Represented is the percentage of viable clones after curcumin treatment respective to untreated cells. Columns, mean of three independent determinations; bars, SD.

The concentration- (2, 4, 6, 8, 10, 20, 30, and 40 μM) and time-related (12 and 24 h) cytotoxicity of curcumin against MCF-7 and MDA-MB-231 cells was assessed using the WST-1 assay. Although similar changes in viability were observed, the sensitivities of both tumor cell lines to curcumin differed (Figure 1). Curcumin was found to be effective at a concentration of 6 μM and to result in a loss of approximately 80% of cell viability at a concentration of 40 μM after a 24 h treatment period in MDA-MB-231 cells (Figure 1A). In contrast, curcumin had no apparent effect, even at a concentration of 10 μM, and led to only an approximately 35% reduction in cell viability at a concentration of 40 μM after a 24 h treatment in MCF-7 cells (Figure 1A). The results revealed that these two different breast cancer cell lines displayed differential susceptibilities to curcumin-induced cytotoxicity.

### The clonogenic ability of MCF-7 and MDA-MB-231 cells differs following curcumin treatment

To further examine the cytotoxic effects of curcumin over a prolonged period of time, clonogenic assays were performed on both of the cell lines 7 or 12 days after treatment with 40 μM curcumin for 3 or 6 h, respectively. DNA analyses of the stained colonies revealed that curcumin treatment for both 3 and 6 h thoroughly abrogated the clonogenic ability of MDA-MB-231 cells (Figure 1B). In MCF-7 cells, however, the same concentration of curcumin did not significantly affect the capacity of the cells to form viable colonies after 3 h of treatment and only caused a slight decrease in the number of colonies after 6 h of treatment (Figure 1B). The results were consistent with the findings in the cell viability assays and further confirmed the differential responses of MCF-7 and MDA-MB-231 cells to curcumin.

### The differential susceptibilities of MCF-7 andMDA-MB-231 cells to curcumin are associated with differential regulation of p27 and p21

**Figure 2:**
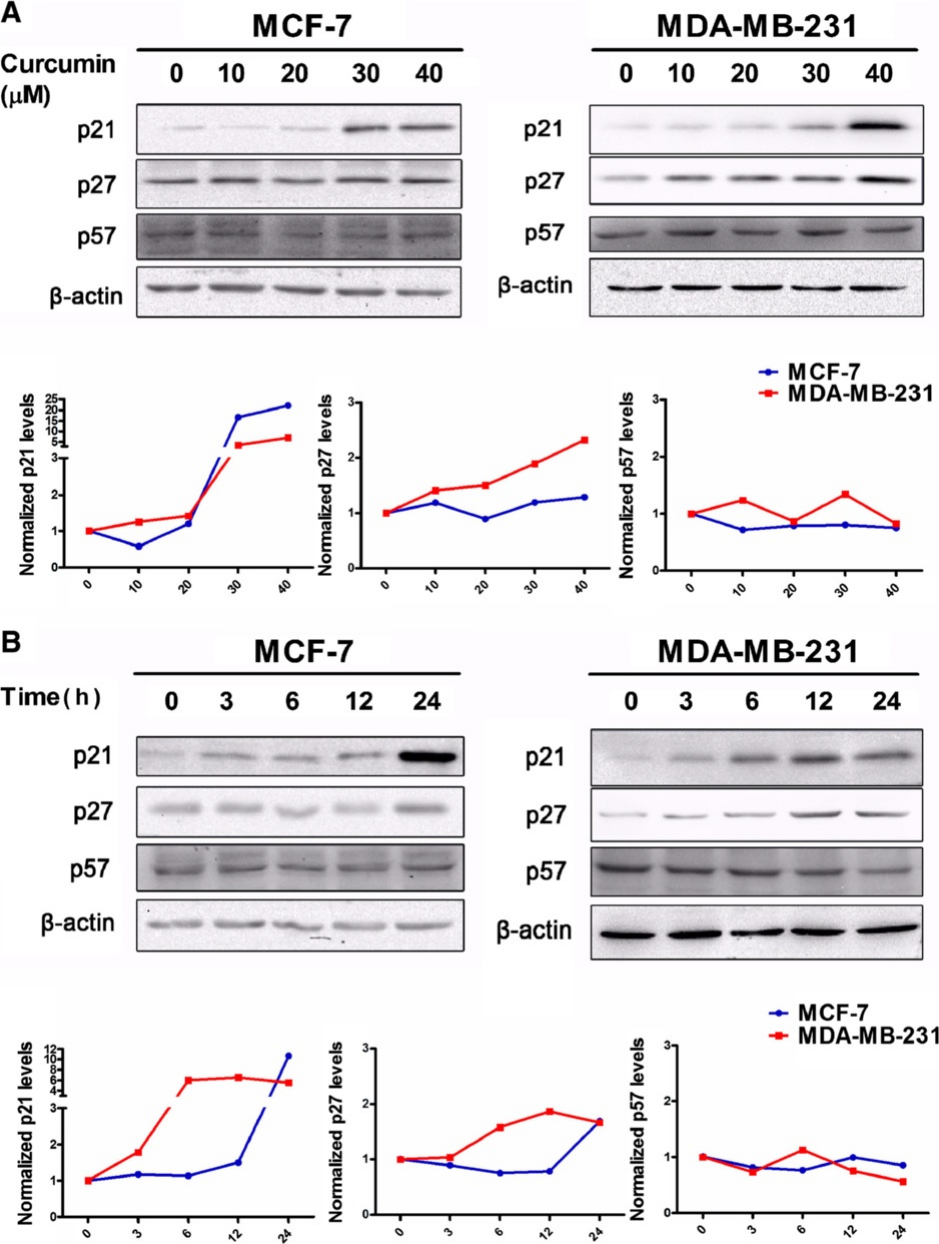
**Analysis of Cip/Kips expression in response to curcumin in MCF-7 and MDA-MB-231 cells. (A)** Analysis of p21 (1:1000), p27 (1:1000) and p57 (1:500) changes induced by indicated concentration of curcumin for 24 h in MCF-7 and MDA-MB-231 cells was took by western blotting. Also shown is a blot for β-actin as a loading control. The representative data of at least two independent experiments are shown. **(B)** Analysis of p21 (1:1000), p27 (1:1000) and p57 (1:500) changes induced by 40 μM curcumin for indicated time in MCF-7 and MDA-MB-231 cells was took by western blotting. Also shown is a blot for β-actin as a loading control. The representative data of at least two independent experiments are shown. Quantitative analysis of Cip/Kips protein expression using Quantity One software (Bio-Rad, USA). Graphs show quantification of Cip/Kips protein expression after normalizing the data to respective control group.

To investigate the possible molecular mechanisms underlying the differential responses of MCF-7 and MDA-MB-231 cells to the cytotoxic effects of curcumin, changes in the expression levels of several important proteins from the CDKi family (i.e., p21, p27 and p57) were examined after curcumin treatment. Marked increases in p27 and p21, which were dependent upon the curcumin doses and incubation times, were observed in the curcumin-sensitive MDA-MB-231 cells (Figure 2). In contrast, the effects of curcumin were less pronounced in the curcumin-resistant MCF-7 cells, and significant p27 and p21 accumulation only occurred at higher concentrations and after longer durations of treatment with curcumin (Figure 2). Unexpectedly, no significant variations in p57 were observable in MCF-7 and MDA-MB-231 cells (Figure 2), suggesting that p57 was not involved in curcumin-induced cytotoxicity. Collectively, these results suggested that the differential cellular responses to curcumin might be closely associated with its differential regulation of p27 and p21, but not p57, in MCF-7 and MDA-MB-231 cells.

### Curcumin-induced down regulation of SKP2 protein and mRNA levels is involved in the induction of p27 and p21

**Figure 3:**
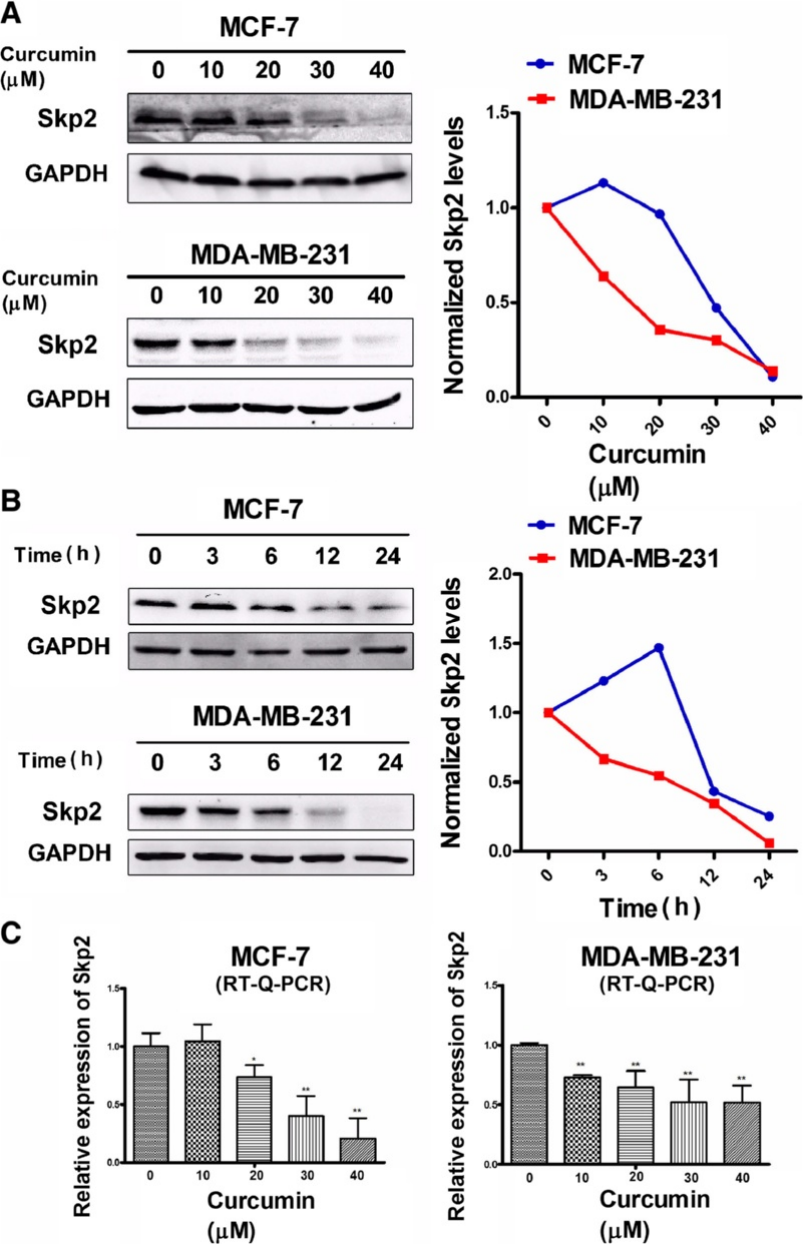
**Analysis of SKP2 at both protein and transcriptional level in response to curcumin. (A)** Analysis of (1:1000) changes induced by indicated curcumin for 24 h in MCF-7 and MDA-MB-231 cells was taken by western blotting. Also shown is a blot for GAPDH as a loading control. The representative data of at least two independent experiments are shown. **(B)** Analysis of SKP2 (1:1000) changes induced by 40 μM curcumin for indicated time in MCF-7 and MDA-MB-231 cells was took by western blotting. Also shown is a blot for GAPDH as a loading control. The representative data of at least two independent experiments are shown. Quantitative analysis of SKP2 protein expression using Quantity One software (Bio-Rad, USA). Graphs show quantification of SKP2 protein expression after normalizing the data to respective control group. **(C)** MCF-7 or MDA-MB-231 cells were treated with indicated concentration of curcumin for 24 h. The bar graph indicates the fold change of SKP2 mRNA level in curcumin treated cells compared with non-treated cells. One-way ANOVA was studied, followed by Student’s t test. Each bar represents the mean ± SD of three independent experiments. *,P <0.05; **,P <0.01, compared with control. RT-Q-PCR, quantitative RT-PCR.

**Figure 4:**
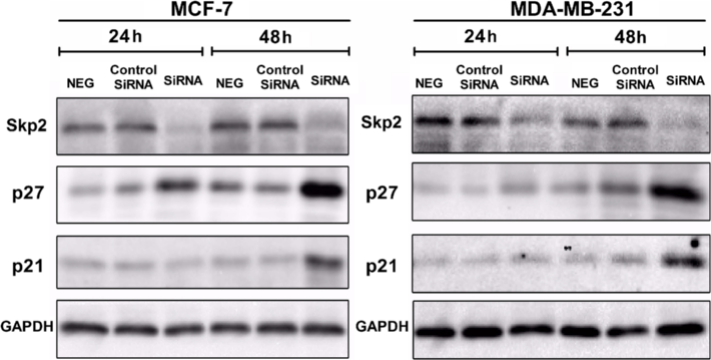
**Knockdown of SKP2 expression in MCF-7 and MDA-MB-231 cells induced up-regulation of p27 and p21.** MCF-7 or MDA-MB-231 cells were transfected with SKP2-SiRNA or negative SiRNA (control-SiRNA) for 24 h or 48 h, (NEG means control group). Down-regulation of SKP2 expression was evaluated by western blotting. A protein level of p21 and p27 in all cells was examined by western blotting. Also shown is a blot for GAPDH as a loading control. The representative data of at least two independent experiments are shown.

Interestingly, the sequential decreases in both SKP2 protein and mRNA levels also differed markedly between the cell lines studied (Figure 3). In MDA-MB-231 cells, suppression of SKP2 was dependent upon the curcumin dose (Figure 3A) and treatment time (Figure 3B). Notably, significant suppression of SKP2 was observed after 3 h and continued through 24 h of curcumin treatment (Figure 3B). In MCF-7 cells, however, significant decreases in SKP2 were only observed with 30 or 40 μM curcumin (Figure 3A) after 12 and 24 h (Figure 3B) of curcumin treatment. In fact, obvious elevations in SKP2 were observed in MCF-7 cells that had been treated with 40 μM curcumin for 3 and 6 h (Figure 3B). In contrast, the QRT-PCR results revealed that curcumin also transcriptionally down-regulated SKP2 in a dose-related manner in both cell types, but curcumin only readily gave rise to an inhibitory effect on SKP2 in MDA-MB-231 cells, even at a lower concentration (10 μM) (Figure 3C). Collectively, these findings revealed that the differential responses of the two cell types to curcumin could be attributed to the differential modulation of p27, p21 and SKP2, and those possible interactions between the induction of p27 and p21 and the suppression of SKP2 may be caused by curcumin treatment. Indeed, it was further demonstrated in the present study that SKP2 gene knockdown in MCF-7 and MDA-MB-231 cells resulted in significant increases in p27 and p21 (Figure 4), supporting the idea that curcumin-mediated SKP2 down-regulation was highly responsible for the induction of p27 and p21.

### Curcumin differentially affects the expression and activity of PKB and its substrates in MCF-7 and MDA-MB-231 cells

**Figure 5:**
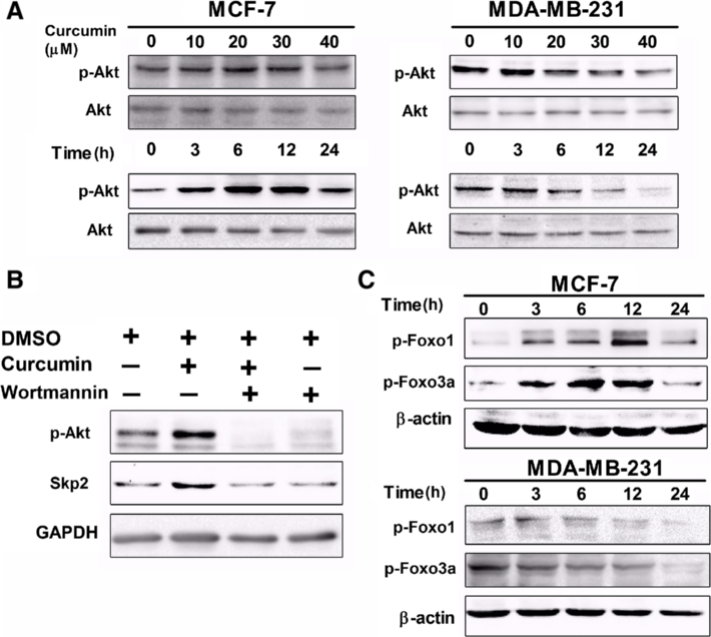
**Curcumin modulated different PI3K/Akt pathway in MCF-7 and MDA-MB-231 cells. (A)** Analysis of p-Akt (1:5000), Akt (1:1000) changes induced by indicated curcumin for 24 h or 40 μM curcumin for indicated time in MCF-7 and MDA-MB-231 cells was took by western blotting. **(B)** MCF-7 cells were treated with 1 μM concentration of wortmannin for 1 h prior to treatment with curcumin (40 μM) for 6 h. Expression of p-Akt (1:5000) and SKP2 (1:1000) in indicated condition was examined by western blotting. Also shown is a blot for GAPDH as a loading control. **(C)** Analysis of p-Foxo1 (1:1000) and p-Foxo3a (1:1000) changes induced by 40 μM curcumin for indicated time in MCF-7 and MDA-MB-231 cells was taken by western blotting. Also shown is blot for β-actin as a loading control. The representative data of at least two independent experiments are shown.

Akt is one of the key determinants of cellular sensitivity to chemotherapeutic drugs and plays a critical role in SKP2 regulation in various cell types at both the transcriptional and post-transcriptional levels [19,25,26]. To examine the interaction between Akt and SKP2, the phosphorylation status of Akt was analyzed in MCF-7 and MDA-MB-231 cells after curcumin treatment. The results indicated that curcumin inhibited the phosphorylation of Akt at Ser-473 in both a dose- and time-related manner (no obvious effects on the expression of total Akt were observed) (Figure 5A), which appeared to be well correlated with the curcumin-induced changes in SKP2 in MDA-MB-231 cells (Figure 3B). In contrast, MCF-7 cells exhibited a more complex pattern of changes in Akt following curcumin treatment (Figure 5A). In this cell line, treatment with lower concentrations of curcumin (10, 20 and 30 μM) for 24 h caused mild increases, while treatment with 40 μM caused a mild decrease, in Akt phosphorylation (Figure 5A). In addition, the time-course (3, 6, 12 and 24 h) of treatment with 40 μM curcumin gave rise to substantial increases in Akt phosphorylation compared to the control in MCF-7 cells, although, notably, at the 24 h time point, 40 μM curcumin readily induced an inhibitory effect on Akt activation compared to that observed at the 3, 6 and 12 h time points (Figure 5A). This pattern of changes in Akt, which was related to the concentration and time of curcumin treatment in MCF-7 cells, was not correlated with the changes in SKP2 (Figures 3A and B), implying the possible involvement of other curcumin-targeted signaling pathways in the modulation of SKP2 in this cell line. Indeed, the combined treatment of MCF-7 cells for 6 h with curcumin and wortmannin, a selective PI3K inhibitor, counteracted both curcumin-induced Akt phosphorylation and SKP2 up-regulation, whereas the use of wortmannin alone or in combination with curcumin did not further augment the down-regulation of SKP2 as compared to the control (Figure 5B). The results thus suggested that curcumin-induced activation of Akt may be an important factor influencing its ability to alter the activity of SKP2, thereby promoting curcumin resistance in MCF-7 cells. Nevertheless, the definitive mechanism underlying the regulation of SKP2 by p-Akt in curcumin-treated MCF-7 and MDA-MB-231 cells requires further study.

In parallel to SKP2, the effects of curcumin on the downstream targets of phospho-Akt, phospho-Foxo3a and phospho-Foxo1 in MCF-7 and MDA-MB-231 cells were also examined. As expected, continuous phosphorylation of Foxo1 at Thr-24 and Foxo3a at Ser-318/321 was observed in MCF-7 cells following curcumin treatment for 3, 6, 12, and 24 h, while in MDA-MB-231 cells, constitutive phosphorylation of Foxo3a and Foxo1 was inhibited by curcumin in a time-dependent manner (Figure 5C). These results suggested the participation of another Akt survival pathway by which MCF-7 cells escape cell death through phosphorylation of FKHR family transcription factors, thus conferring curcumin resistance.

### Treatment with a PKB inhibitor and SKP2 siRNA sensitizes breast cancer cells to curcumin

**Figure 6:**
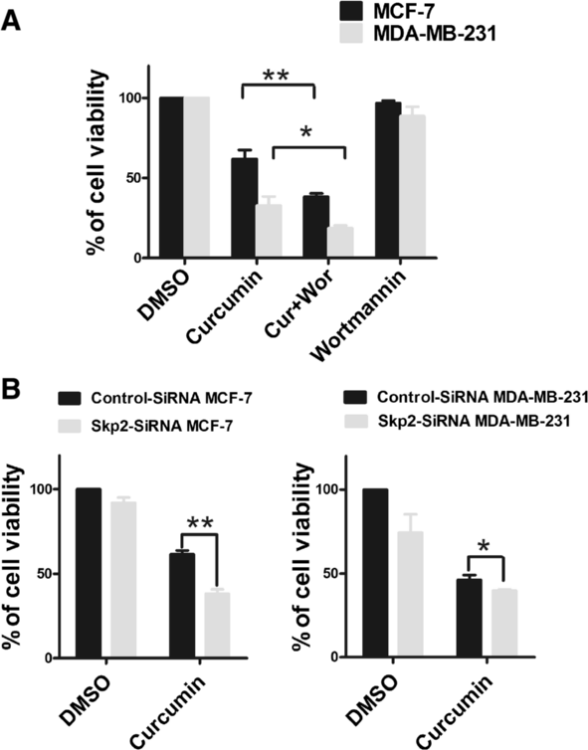
**Cell viability assay for treatment of curcumin with PKB inhibitor or SKP2 SiRNA. (A)** Wortmannin alters curcumin response in MCF-7 and MDA-MB-231 cells. MCF-7 and MDA-MB-231 cells were treated with 1 μM concentration of wortmannin for 1 h prior to treatment with curcumin (40 μM) for 12 h and WST-1 assay was performed as described in the “Materials and methods”. Each bar represents the mean ± SD of three independent experiments. *,P <0.05; **,P <0.01, compared with control (DMSO). **(B)** Silencing of SKP2 increases cellular sensitivity to curcumin in MCF-7 and MDA-MB-231 cells. Cells were seeded at a density of 5000 cells/well in 96-well plate and cultured overnight. Then the medium was replaced by 1%(V/V) FBS, 20nM SKP2-SiRNA and control-SiRNA was added to indicated wells and cells were cultured for 48 h. SKP2-SiRNA transfection cells and control-SiRNA transfection cells were obtained. Cells were treated with 40 μM curcumin for 12 h and WST-1 assay was performed to detect viability of cells. One-way ANOVA was studied, followed by Student’s t test. Each bar represents the mean ± SD of three independent experiments. *,P <0.05; **,P <0.01, compared with control (DMSO).

To further explore whether the modulation of the Akt/SKP2 signaling axis would eventually lead to phenotypic changes in cellular susceptibilities to the cytotoxicity of curcumin, viability assays on MCF-7 and MDA-MB-231 cells were again performed. Co-treatment with curcumin and wortmannin revealed that the use of the Akt inhibitor not only synergistically enhanced curcumin-induced cytotoxicity in both cell lines but also rendered MCF-7 cells more susceptible to curcumin, similar to MDA-MB-231 cells (Figure 6A). Similarly, SKP2 knockdown also led to the enhancement of curcumin toxicity in MCF-7 and MDA-MB-231 cells (Figure 6B). Notably, the loss of cell viability in SKP2-siRNA transfected MCF-7 cells was 1.5-fold higher (63%) than that observed (38%) in the control-siRNA transfected cells (Figure 6B), strongly suggesting that curcumin-induced SKP2 up-regulation plays an important role in resistance to cell death in MCF-7 cells. Collectively, these results indicated that the differential susceptibilities to the cytotoxicity of curcumin that were observed in MCF-7 and MDA-MB-231 cells were highly attributable to the differential modulatory activity of curcumin on Akt/SKP2 signaling and that concomitant treatment with inhibitors of the Akt/SKP2 pathway sensitized both MDA-MB-231 and curcumin-resistant MCF-7 cells to curcumin treatment.

## Discussion

Cip/Kips family proteins, such as p21 and p27, are well documented to function as both sensors and effectors of multiple anti-proliferative signals, and the absence of p21 and p27 has been linked to drug resistance in multiple human malignancies [27-29]. In breast cancer cells specifically, multi-phytochemical induced cytotoxicity has been revealed to be highly associated with p27 or p21 induction [30-32]. For instance, p21 and p27 are greatly enhanced following curcumin (50 μM) treatment for 3 h in MCF-7 cells [33]. Chiu et al. also demonstrated that curcumin increased p21 independently of p53 in MDA-MB-231 cells [34]. Similarly, while the results of our study demonstrated the effects of curcumin on p21 and p27 in MCF-7 and MDA-MB-231 cells, we further revealed, for the first time, that curcumin altered p27 and p21 in concentration- and time-related manners (Figure 2), which was in agreement with the differential response patterns of the two cell types to the cytotoxicity of curcumin (Figure 1). The curcumin-mediated functional changes in p21 and p27 may thus provide a molecular explanation as to why MCF-7 cells were resistant, while MDA-MB-231 cells were sensitive, to the cytotoxic effects of curcumin, which was also supported by recent findings indicating that the dual knockdown of the p21 and p27 genes lead to a significant suppression of MCF-7 cell proliferation. However, it is worth noting that our findings differed from an earlier report by Shao et al., in which treatment with higher concentrations of curcumin (up to 50 μM) for 72 h did not cause variations in p21, although multiple inhibitory effects were observed after curcumin (50 μM) treatment for 4 days in both MCF-7 and MDA-MB-231 cells [35]. This discrepancy may be explained by the fact that the high curcumin concentration (50 μM) and prolonged treatment time (72 h) that was used in their study have resulted in extensive cell death.

Although we demonstrated in the present study that the distinct changes in p21 and p27 in MCF-7 and MDA-MB-231 cells may be linked to the differential susceptibilities of the cell lines to the effects of curcumin, the upstream signaling that affected p21 and p27 still remained elusive. Given that SKP2 plays critical roles in mediating the ubiquitin-dependent degradation of some cell-cycle proteins, including p27, p21 and p57 [12,13], and that aberrant SKP2 expression may contribute to the progression and development of a number of malignant tumors in humans, the effects of curcumin on SKP2 and the molecular relationship between these effects and the differential induction of p27 and p21 were further investigated in MCF-7 and MDA-MB-231 cells. We found that in the curcumin-sensitive MDA-MB-231 cells, curcumin significantly downregulated the expression of SKP2 at both the protein and transcriptional levels (Figures 3A and C). In the curcumin resistant MCF-7 cells, however, the suppression of SKP2 was only observed at higher concentrations (30 or 40 μM) and after a longer duration (12 or 24 h) of curcumin treatment (Figure 3B). In addition, a significant increase in SKP2 appeared following 3 or 6 h curcumin treatment (40 μM) (Figure 3B), indicating a deregulation of this protein in this cell line. Such distinct patterns of SKP2 in response to curcumin treatment were not only well correlated with the differential cytotoxicity but also with the alterations in p21 and p27 that were observed in MCF-7 and MDA-MB-231 cells, suggesting an interaction between SKP2 and p21/p27 that functionally governed the susceptibility of the two different cell types to curcumin. This interaction was further confirmed by the SKP2 siRNA assay, which revealed that SKP2 knockdown led to significant elevations in p21 and p27 expression in both MDA-MB-231 andMCF-7 cells (Figure 4), suggesting a mechanistic dependency of the curcumin-induced elevations of p21 and p27 on the suppression of SKP2. Previous studies have shown that antisense oligonucleotides against SKP2 mRNA inhibited the proliferation of cancer cell lines and increased the sensitivity of these cells to apoptotic stimuli [36,37]. In particular, knockdown of SKP2 in MCF-7 cells inhibited cell growth and enhanced the cytotoxic effects of epirubicin [38]. In addition, several studies revealed that the defective regulation of SKP2 was the key underlying cause of rapamycin resistance in multiple tumors and that SKP2 levels are a key determinant of antitumor responses to mTOR inhibitors [39]. While in line with these observations, our findings further suggest that the differential susceptibilities to the cytotoxic effects of curcumin may be highly associated with the SKP2-Cip/Kips (p21 and p27) pathway, as observed in both cell lines, and that the deregulation of SKP2 and p21/p27 signaling may be responsible for curcumin resistance, as observed in MCF-7 cells. Furthermore, while a number of studies have focused on quantitative changes, such as the fact that the low levels of p27 in breast cancer cells were due to the high expression of SKP2 [14,40], very little attention has been paid to the relationship between p21 and p57 and SKP2-mediated degradation in breast cancer cells. The data from the present study further suggest a new possible mechanism underlying curcumin-induced changes in SKP2expression in MCF-7 and MDA-MB-231 cells that may be an important determinant ofp21 and p27 stability by reducing the rate of their SKP2-mediated degradation. In addition, no changes in p57 were observed in MCF-7 and MDA-MB-231 cells following curcumin treatment(Figure 2), regardless of SKP2 knockdown (data not shown), suggesting that the degradation of p57 in these cell lines may occur independently of SKP2, which differs from that observed in other types of tumors [41]. Therefore, the extent of the functional dependency on SKP2 of different Cip/Kips proteins (p27, p21, and p57) in different breast cancer subtypes requires further study.

The constitutive or increased activity of the PI3K/Akt-dependent signaling cascade presents a major means by which cancer cells achieve uncontrolled proliferation, invasion, angiogenesis and drug resistance [42,43]. Akt signaling has also been reported to play a critical role in SKP2 regulation in various cell types at both the transcriptional and post-translational levels [19,25,27]. To examine the interaction between Akt and SKP2, the phosphorylation status of Akt inMCF-7 and MDA-MB-231 cells after curcumin treatment was analyzed in this study. We demonstrated that in MDA-MB-231 cells curcumin could markedly inhibit the phosphorylation of Akt at Ser-473 in both a dose- and time-dependent manner (Figure 5A), which was in agreement with the changes that we observed in the expression pattern of SKP2 (Figures 3A and B), indicating that the inhibitory effects of curcumin on SKP2 may be highly related to the inhibitory role of curcumin in Akt activation. In MCF-7 cells, however, curcumin treatment caused a substantial increase in Akt phosphorylation, which exhibited a pattern that substantially differed from that of Akt in MDA-MB-231 cells (Figure 5A). Moreover, Akt phosphorylation at Ser-473 was unexpectedly observed to be maintained at high levels over the various curcumin concentrations or treatment times in MCF-7 cells (Figure 5A), which did not correspond to the curcumin-induced changes inSKP2 (Figures 3A and B). The present results demonstrated that curcumin treatment with either a lower concentration (10 or 20 μM) or a higher concentration (40 μM) for a shorter period of time (3 or 6 h) resulted in SKP2 up-regulation that could be attributed to Akt activation (Figures 3A, B and 5A). These results were confirmed by the co-administration of curcumin with the selective PI3K inhibitor, wortmannin, which reversed the phosphorylation of Akt and the up-regulation of SKP2 in MCF-7 cells (Figure 5B). In contrast, when a higher concentration of curcumin (30 or 40 μM) was used, the resulting up-regulationof SKP2didnot occur in response to the elevated Akt phosphorylation (Figures 3A, B and 5A), implying the involvement of another curcumin-targeted signaling pathway, rather than merely Akt, which predominantly modulates SKP2 expression. Indeed, recent evidence has indicated a critical role of Focal Adhesion Kinase (FAK) in the regulation of Cip/Kips through SKP2-dependent and SKP2-independent mechanisms [44,45]. Whether the FAK-associated pathway is a direct target of curcumin requires further investigation. With regard to the interaction between Akt and SKP2 in breast cancer cell lines, several previous studies have obtained data that were similar to our results, in which the treatment of MCF-7 cells with inhibitor of PI3K (LY294002) was shown to significantly decrease the expression of SKP2 [24], and the use of rapamycin, an mTOR inhibitor, induced cytotoxicity via the suppression of SKP2 in MDA-MB-231 cells [46]. The present study further explored the idea that the distinct behavior of the PI3K/Akt-SKP2 signaling pathway in response to curcumin was highly associated with the differential susceptibilities of MCF-7 and MDA-MB-231 cells to the cytotoxic effects of curcumin, in which the levels of Akt phosphorylation was an important factor influencing its ability to determine the activity of SKP2 and thereby affected the responses of the breast cancer cells to curcumin. This notion was validated by the results of the WST-1 assay, which indicated that the use of a PI3K inhibitor (wortmannin) or the down-regulation of SKP2 (SKP2 siRNA) not only augmented the sensitivity of the curcumin-sensitive MDA-MB-231 cells, but more impressively, also significantly enhanced the response of the curcumin-resistant MCF-7 cells to curcumin treatment compared to curcumin alone (Figure 6). Despite the fact that the exact mechanism underlying the curcumin-mediated regulation of SKP2 is not fully understood, the present study illuminated the fact that the PI3K/Akt-SKP2 pathway functioned differentially in response to curcumin in the two breast cancer subtypes and that the ability of curcumin to inactivate SKP2-dependent signaling was impaired by the continuous Akt phosphorylation in the curcumin-resistant MCF-7 cells, suggesting that the co-administration of curcumin and PI3K- and/ or SKP2-targeted inhibitors may be better alternatives in clinical practice.

In addition to SKP2, the FKHR family transcription factors (FOXO) have also been reported to be downstream targets of Akt that mediate apoptosis in breast cancer [47]. FOXO activation promoted the transcription of genes involved in cell cycle arrest and apoptosis [48]. One mechanism by which Akt promotes cell survival is via FOXO phosphorylation, which inactivates FOXO and prevents apoptosis [49,50]. Interestingly, we also found that both Foxo1 and Foxo3a responded in different manners to curcumin treatment in MCF-7 and MDA-MB-231 cells. Curcumin treatment caused Foxo1 and Foxo3a dephosphorylation in MDA-MB-231 cells, while Foxo1 and Foxo3a continued to be phosphorylated in MCF-7 cells (Figure 5C). Notably, the patterns of Foxo1 and Foxo3a changes that were brought about by curcumin treatment were in agreement with those observed for p-Akt in MCF-7 and MDA-MB-231 cells, suggesting that another Akt survival pathway modulated the phosphorylation status of FOXO, which may also explain why MDA-MB-231 cells were sensitive to curcumin treatment, while MCF-7 cells were resistant.

## Conclusions

Our study established the importance of the PI3K/Akt-SKP2-Cip/Kips signaling pathway and revealed that the discrepant modulation of this pathway by curcumin was highly responsible for the differential susceptibilities of these two cell types to curcumin. Accordingly, the results of the present study further suggested that p-Akt and SKP2 are both important pharmacogenomic markers that can be used to predict the sensitivity of breast cancer cells to curcumin and that SKP2 silencing and p-Akt inhibition may be potent therapeutic alternatives that can be used in combination with curcumin to treat different breast cancer subtypes.

## Methods

### Chemicals and reagents

Curcumin, with a purity ≥95%, was obtained from Unilever R&D (Sanjivani Phytopharma Pvt. Ltd, India). Wortmannin and dimethyl sulfoxide (DMSO) were purchased from Sigma Chemical Co. (St. Louis, MO). DMEM GlutaMAX medium containing L-Glutamine, and Leibovitz’s L-15 medium containing L-Glutamine and 0.05% Trypsin-EDTA, were obtained from Gibco BRL (Grand Island, NY, USA). FBS was obtained from PAA (Coelbe, Germany). Antibodies that had been raised against S-phase kinase associated protein 2 (SKP2), p27 Kip1, p21 Waf1/Cip1, phospho-Foxo1 (Thr-24), p-Foxo3a (Ser-318/321), β-actin, GAPDH and an anti-rabbit IgG antibody linked with HRP were purchased from Cell Signaling Technology (Beverly, MA). Antibodies that had been raised against Akt and p57 were obtained from Bioworld (China). An antibody that had been raised against p-Akt Ser-473 was obtained from Abcam (Cambridge, UK).

### Cell culture and curcumin treatment

MCF-7 cells were obtained from the American Type Culture Collection (ATCC, Rockville, MD). MDA-MB-231 cells were purchased from Cell Bank of the Chinese Academy of Sciences (Shanghai, China), where the cells were characterized by mycoplasma detection, DNA –Fingerprinting, isoenzyme analysisand cell vitality detection. All these cells were maintained in American Type Culture Collection recommended cell culture media andconditions. MCF-7 cells were grown in DMEM medium containing 10% (V/V) FBS without antibiotics at 37°C in a humidified atmosphere containing 95% air and 5% CO_2_. MDA-MB-231 cells were grown in L-15 medium containing 10% (V/V) FBS without antibiotics at 37°C. Curcumin was dissolved in DMSO to a stock concentration of 20 mM in a dark colored bottle at −20°C and was diluted to the required concentration with medium when needed. Prior to curcumin treatment, the cells were grown to a density of approximately 80% and were then treated with curcumin at different concentrations for the indicated times. The control cells were incubated with DMSO at the same final concentration.

### Cell viability assay

Cell viability was determined in vitro using the water-soluble tetrazolium WST-1 assay (Roche Molecular Biochemicals, Mannheim, Germany). The cells were seeded at a density of 10,000 cells/ well in 96 well plates and cultured overnight. The cells were then treated with curcumin at various concentrations (2, 4, 6, 8, 10, 20, 30, and 40 μM) for 12 and 24 hours. Cells that were incubated with an equivalent amount of DMSO without curcumin served as controls. After the treatments, the medium was aspirated and the cells were washed with PBS. Then, 10 μl of WST-1 reagent was added to each well. The plates were mixed gently and the cells were incubated for 2 to 4 hours. After the incubation period, the plates were mixed gently on an orbital shaker for one minute and the absorbance of each sample was measured at 430 nm using a SpectraMax M5 plate reader (Molecular Devices, Sunnyvale, CA).

### Clonogenic assay

The breast cancer cells were plated in six-well plates overnight and treated with 40 μM curcumin for a period of 3 or 6 h. After the removal of the drug-containing medium, the cells were washed using PBS, trypsinized and plated at a low density (2000 cells/ well in six-well plates). The cells were then incubated with an equivalent amount of DMSO without curcumin, which served as a control. The cells were cultivated for 7 or 12d and the medium was refreshed every two days. The colonies were stained with crystal violet (Sigma Chemical Co, St. Louis, MO). The number of clones in a given area was counted for each condition.

### Quantitative real-time reverse transcription-PCR

Total RNA was extracted using Trizol reagent (Invitrogen, USA). Two micrograms of RNA was reverse transcribed using the Reverse Transcription System (Promega, USA), according to the manufacturer’s recommended instructions. The cDNA was diluted ten-fold prior to PCR amplification. Real-time PCR was performed using a FastSYBR Mixture (CWBIO, China). The primer pairs that were used were as follows: SKP2, (forward) 5′-GCTGCTAAAGGTCTCTGGTGT-3′, (reverse) 5′- AGGCTTAGATTCTGCAACTTG-3′; and GAPDH, which served as an internal control, (forward) 5′-TCTCTGCTCCTCCTGTTC-3′ and (reverse) 5′-CTCCTGGAAGATGGTGATG-3′. The mRNA levels of SKP2 were quantified by measuring the threshold cycle (Ct).

### Western blotting

The cells were treated as described in the figure legends. After the treatments, the cells were placed on ice, washed with cold PBS and lysed in RIPA lysis buffer (Beyotime, China) that had been supplemented with 1 mM PMSF, a protease inhibitor cocktail and a phosphatase inhibitor cocktail (KangChen, China). Cell lysates were centrifuged at 14,000 × *g* at 4°C for 5 mins. Protein concentrations were determined using a BCA kit (Beyotime, China). Approximately 40–50 μg of cellular protein from each sample was loaded onto 8% or 12% SDS-polyacrylamide gels and electrotransferred to nitrocellulose (NC) membranes (0.22/0.45 μm) (Millipore Billerica, USA). The blotted membranes were incubated with different primary antibodies, followed by incubations with secondary antibodies. The proteins were visualized using an ECL kit (Millipore, Billerica, USA). Relative protein levels were normalized to β-actin/GAPDH. A Quantity One Gel Doc XR gel imaging system (Bio-Rad, USA) was used for the detection and analysis of band intensity.

### Specific RNA-mediated SKP2 down-regulation

Small interfering RNAs (siRNAs) targeting SKP2 were purchased from Qiagen. For transient expression, the cell lines were transfected using HiPerFect Transfection Reagent (Qiagen), as outlined in the HiPerFect Transfection Reagent Handbook (Fifth edition, Qiagen). AllStars Negative siRNA AF 488 (Qiagen) was used both as a negative control and to determine the transfection efficiency. Two independent experiments were performed. SKP2 siRNA data: the target sequence is 5′-AAGTGATAGTCATGCTAAA-3′, the sense strand is 5′-GUGAUAGUGUCAUGCUAAATT-3′ and the antisense strand is 5′-UUUAGCAUGACACUAUCACTT-3′.

### Statistical analyses

The values were expressed as the means ± SD. The statistical significance of the mean values among the different cell lines was determined using one-way ANOVA, followed by Student’s t test.
